# Association Between Sleep Duration and Cognitive Frailty in Older Chinese Adults: Prospective Cohort Study

**DOI:** 10.2196/65183

**Published:** 2025-04-23

**Authors:** Ruixue Cai, Jianqian Chao, Chenlu Gao, Lei Gao, Kun Hu, Peng Li

**Affiliations:** 1 Department of Epidemiology and Health Statistics, School of Public Health, Southeast University, Nanjing, China; 2 Department of Anesthesia, Critical Care and Pain Medicine, Massachusetts General Hospital, 149 13th Street, Boston, MA, 02129, United States, 1 6176516591; 3 Division of Sleep Medicine, Harvard Medical School, Boston, MA, United States; 4 Division of Sleep and Circadian Disorders, Departments of Neurology and Medicine, Brigham and Women’s Hospital, Boston, MA, United States; 5 Broad Institute of MIT and Harvard, Cambridge, MA, United States

**Keywords:** aging, frailty, cognition, cohort study, sleep duration, sleep quality, longitudinal study

## Abstract

**Background:**

Disturbed sleep patterns are common among older adults and may contribute to cognitive and physical declines. However, evidence for the relationship between sleep duration and cognitive frailty, a concept combining physical frailty and cognitive impairment in older adults, is lacking.

**Objective:**

This study aimed to examine the associations of sleep duration and its changes with cognitive frailty.

**Methods:**

We analyzed data from the 2008‐2018 waves of the Chinese Longitudinal Healthy Longevity Survey. Cognitive frailty was rendered based on the modified Fried frailty phenotype and Mini-Mental State Examination. Sleep duration was categorized as short (<6 h), moderate (6‐9 h), and long (>9 h). We examined the association of sleep duration with cognitive frailty status at baseline using logistic regressions and with the future incidence of cognitive frailty using Cox proportional hazards models. Restricted cubic splines were used to explore potential nonlinear associations.

**Results:**

Among 11,303 participants, 1298 (11.5%) had cognitive frailty at baseline. Compared to participants who had moderate sleep duration, the odds of having cognitive frailty were higher in those with long sleep duration (odds ratio 1.71, 95% CI 1.48‐1.97; *P*<.001). A J-shaped association between sleep duration and cognitive frailty was also observed (*P*<.001). Additionally, during a mean follow-up of 6.7 (SD 2.6) years among 5201 participants who were not cognitively frail at baseline, 521 (10%) participants developed cognitive frailty. A higher risk of cognitive frailty was observed in participants with long sleep duration (hazard ratio 1.32, 95% CI 1.07‐1.62; *P*=.008).

**Conclusions:**

Long sleep duration was associated with cognitive frailly in older Chinese adults. These findings provide insights into the relationship between sleep duration and cognitive frailty, with potential implications for public health policies and clinical practice.

## Introduction

Physical frailty and cognitive impairment are prevalent among older adults and have individually been associated with adverse health outcomes [1,2]. They often coincide with aging and can be bidirectionally linked to each other [3,4], prompting the introduction of the concept of cognitive frailty—the coexistence of both physical frailty and cognitive impairment [5]. The necessity is further justified by the findings that cognitive frailty poses an even greater risk of adverse outcomes compared to the isolated effects of the 2 conditions [6,7]. Identifying factors associated with or contributing to cognitive frailty is important and will provide a better understanding of the underlying mechanisms.

Disturbed sleep patterns, such as an increased number of awakenings, abnormal nighttime sleep duration, and poorer sleep quality, are common among older adults [8]. Numerous epidemiological studies identified sleep disturbances as risk factors for physical frailty and cognitive impairment, such as shorter or longer sleep duration, insomnia, and sleep apnea [9,10]. In addition, recent studies have linked long sleep duration to cognitive frailty, but they are from cross-sectional analyses [11,12]. A longitudinal study in Mexican older adults reported associations between increasing sleep duration trajectory with greater odds for mild cognitive impairment and frailty separately [13]. Whether sleep patterns are associated with the development of cognitive frailty as a unified concept remains to be elucidated.

To address this knowledge gap, this study aimed to assess the associations of sleep duration with cognitive frailty in a nationally representative, large cohort of older Chinese adults. By focusing on an older Chinese adult population, our findings may offer insights into the relationship between sleep duration and cognitive frailty within this demographic, with potential implications for public health policies and clinical practice.

## Methods

### Study Design and Participants

We used data from the Chinese Longitudinal Healthy Longevity Survey (CLHLS). As detailed previously, CLHLS is an ongoing, nationwide representative longitudinal cohort study of Chinese adults aged 65 years and older from 23 out of 31 provinces or municipalities or autonomous regions in mainland China [14,15]. The CLHLS started in 1998 with surveys on sociodemographic characteristics, lifestyle, cognitive function, psychological status, and physical function conducted every 2‐3 years.

In this study, we used data from 4 waves (2008, 2011, 2014, and 2018) of the CLHLS. To maximize statistical power and leverage the large sample size, we first examined cross-sectional associations between sleep duration and sleep quality, with cognitive frailty at baseline (ie, the 2008 wave). For this set of analyses, we included 11,303 participants who had cognition and physical frailty assessments at baseline. We then examined whether baseline sleep duration and sleep quality, as well as change in sleep duration, were associated with the future development of cognitive frailty. For these longitudinal analyses, we excluded 1298 participants who had cognitive frailty at baseline, 4780 participants who had no follow-up cognitive frailty assessment, and 24 participants who had missing value in sleep duration in the 2011 wave. As a result, 5201 participants entered the subsequent longitudinal analysis.

### Assessment of Cognitive Frailty

Cognitive frailty was identified as the simultaneous presence of both physical frailty and cognitive impairment [5].

Physical frailty was assessed by a modified Fried frailty phenotype, based on 5 components: shrinking, weakness, low mobility, exhaustion, and inactivity [16,17]. Shrinking was defined as having a BMI of 18.5 kg/m^2^ or less. Weakness was determined if participants self-reported that they were unable to lift a bag weighing 5 kg or above. Low mobility was identified if participants self-reported that they were unable to walk 1 km or longer in a row. Exhaustion was identified if participants answered “always,” “often,” or “sometimes” to the question “Do you feel the older you get, the more useless you are?” Inactivity was defined as participants doing the following activities 1 time per week or less: housework, outside activity, garden work, raising domestic animals or pets, playing cards or mah-jongg, and social activity (organized). Participants were considered physically frail when meeting ≥3 of the 5 criteria.

Cognitive impairment was assessed by a validated Chinese version of the Mini-Mental State Examination (C-MMSE) [15,18]. The C-MMSE includes 24 items regarding orientation, memory, attention, calculation, and language, ranging from 0 to 30. Based on a prior study, participants were considered cognitive impairment if the C-MMSE score was ≤22 [15].

### Assessment of Sleep Variables

Sleep duration was estimated based on the answers to the question “How many hours do you sleep normally?” Based on their answers, participants were further categorized into short (<6 h), moderate (6‐9 h), and long (>9 h) sleep durations. This categorization aligned with previous research indicating that both short and long sleep durations are associated with poorer health outcomes [19]. The rate of change in sleep duration was calculated by taking the difference between sleep durations reported in the 2008 and 2011 waves (ie, sleep duration in the 2011 wave minus sleep duration in the 2008 wave) and dividing it by the individual follow-up interval in years. Sleep quality was assessed based on answers to the question “How about the quality of your sleep?” A score of “poor” was assigned if the answer was “so-so,” “bad,” or “very bad,” and a score of “good” was assigned otherwise.

### Covariates

We considered the following covariates that have been identified to be associated with physical or cognitive outcomes in previous studies [20,21]: age, sex, education, marital status, residence, economic status, loneliness, smoking status, drinking status, and multimorbidity. Education was categorized as “Not Educated” if the participant had not attended any schools and “With Formal Education” if the participant had attended schools more than 1 year. Marital status was defined as currently married and living with a spouse, and others (eg, separated, divorced, widowed, and never married). Residence was categorized into living in urban (eg, city or town) or rural areas. Economic status was categorized into economic dependence or independence based on their income source. Loneliness was identified if participants answered “always,” “often,” or “sometimes” to the question “Do you feel lonely and isolated?” Smoking status was categorized as “Never smoked,” and “Former or current smoker.” Drinking status was categorized into “Never drank,” and “Former or current drinker.” Multimorbidity was defined from self-reports and categorized as “Yes” if having 2 or more of the following chronic diseases: hypertension, diabetes, heart disease, stroke or cerebrovascular disease, respiratory disease, cancer, and Parkinson disease, or “No” if otherwise.

### Statistical Analysis

Listwise deletion was applied to both cross-sectional and longitudinal analyses to handle missing data on covariates. The *t* tests for continuous variables and chi-square tests for categorical variables were used to compare the differences in the baseline characteristics.

Multivariate logistic regression models were performed to assess the associations of sleep duration and sleep quality with cognitive frailty at baseline (ie, the 2008 wave). We performed 3 models: model A was unadjusted; model B was adjusted for age, sex, and education; model C was further adjusted for marital status, residence, economic status, loneliness, smoking status, drinking status, and multimorbidity.

Cox proportional hazard (PH) models were used to test the associations between sleep duration and sleep quality with incident cognitive frailty during the follow up. Death was treated as a right-censored event at the time of occurrence. The follow-up duration was defined as the time interval in years from baseline to the date of the first occurrence of cognitive frailty, death, or the latest available data, whichever occurred first. We rounded the time to the nearest integer year to account for group-tied events. Similarly, we performed 3 models: model A was unadjusted; model B was adjusted for age, sex, and education; model C was further adjusted for marital status, residence, economic status, loneliness, smoking status, drinking status, and multimorbidity. The PH assumption of the Cox models was assessed using Schoenfeld residuals. No major violations of the PH assumption were detected for exposure variables.

Restricted cubic spline (RCS) curves were plotted to explore the nonlinear cross-sectional and longitudinal associations between sleep duration and cognitive frailty. Additionally, several sensitivity analyses were conducted to verify the robustness of our findings. First, we adjusted for each chronic disease individually and for all chronic diseases simultaneously. Second, we performed stratified analyses by sex and age. Furthermore, we performed similar sets of Cox PH models to investigate the associations of changes in sleep duration and incident cognitive frailty.

All statistical analyses were performed in R (version 4.1.2; R Core Team). Statistical significance was considered at a 2-tailed ɑ level of .05.

### Ethical Considerations

The CLHLS was approved by the Duke University Institutional Review Board (Pro00062871) and the Peking University Biomedical Ethics Committee (IRB00001052–13074). All participants provided written informed consent. The data used in this study were deidentified to protect participant privacy and confidentiality. No compensation was provided to participants.

## Results

### Participant Characteristics

**Figure 1.**
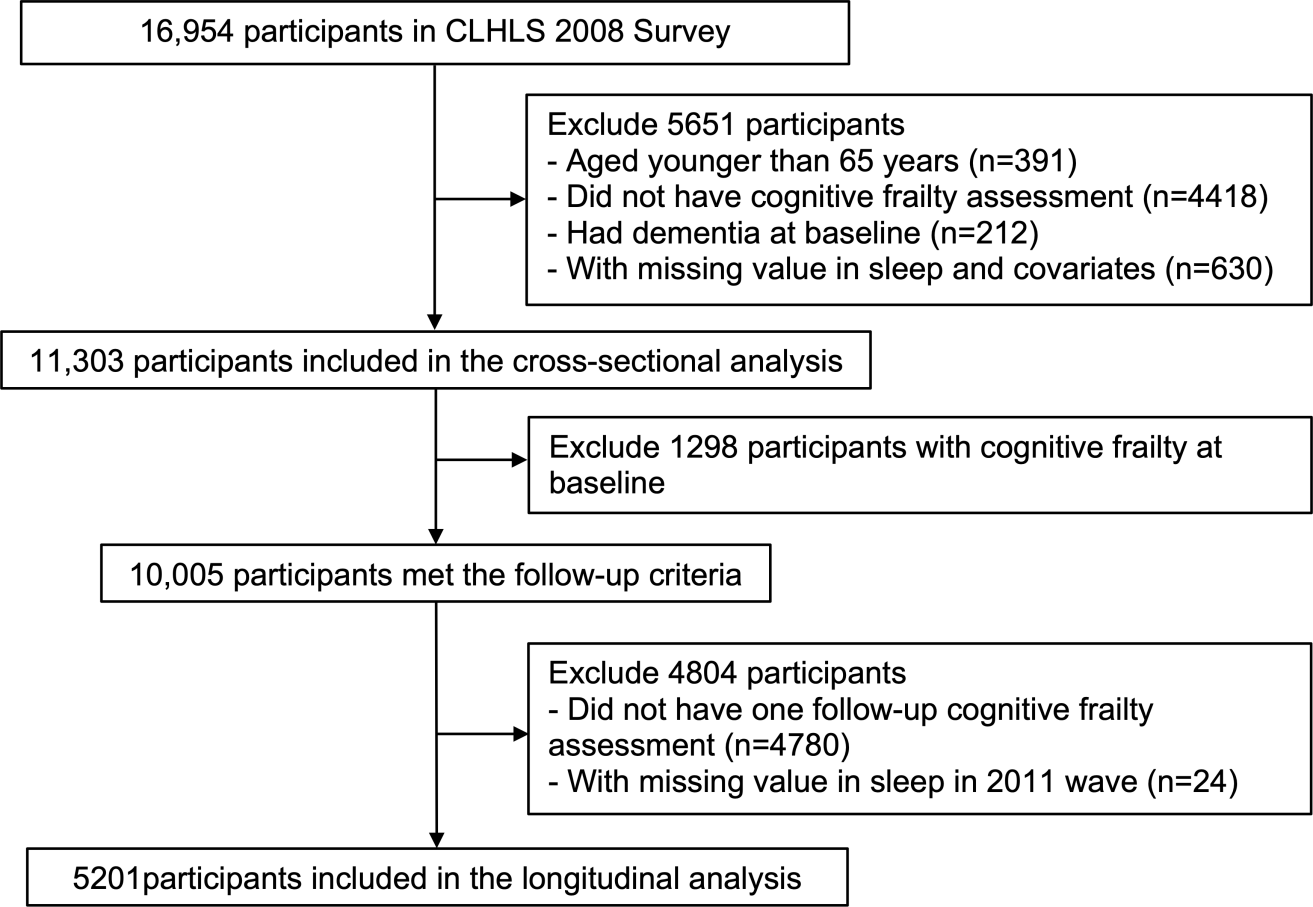
Flowchart of participants through the study. CLHLS: Chinese Longitudinal Healthy Longevity Survey.

The participant flowchart is presented in Figure 1. Of the 11,303 participants included in the cross-sectional analysis, 6037 (53.4%) participants were female. The mean age at baseline was 84.7 (SD 11.0) years old. Approximately 11.5% (n=1298) of all participants had cognitive frailty at baseline. The numbers of participants who reported short, moderate, or long sleep duration were 1327 (11.7%), 7207 (63.8%), and 2769 (24.5%), respectively. Poor sleep quality was found in 3796 (33.6%) participants. Baseline characteristics stratified by cognitive frailty status at baseline are summarized in Table 1. Additionally, baseline characteristics categorized by sleep duration are presented in Multimedia Appendix 1. Baseline characteristics of 5201 participants included in the longitudinal analysis are summarized in Multimedia Appendix 2.

**Table 1.** Baseline characteristics of participants by cognitive frailty status at baseline.

	Overall (n=11,303)	Non-CF^a^ (n=10,005)	CF (n=1298)	*P* value
Age (years), mean (SD)	84.7 (11.0)	83.3 (10.6)	95.3 (7.2)	<.001
Sex, n (%)				<.001
Male	5266 (46.6)	4966 (49.6)	300 (23.1)	
Female	6037 (53.4)	5039 (50.4)	998 (76.9)	
Education, n (%)				<.001
Not educated	6548 (57.9)	5457 (54.5)	1091 (84.1)	
With formal education	4755 (42.1)	4548 (45.5)	207 (15.9)	
Marital status, n (%)				<.001
Married and living with spouse	4098 (36.3)	3979 (39.8)	119 (9.2)	
Others	7205 (63.7)	6026 (60.2)	1179 (90.8)	
Current residence, n (%)				.12
Urban	4647 (41.1)	4140 (41.4)	507 (39.1)	
Rural	6656 (58.9)	5865 (58.6)	791 (60.9)	
Economic status, n (%)				<.001
Dependence	7929 (70.2)	6716 (67.1)	1213 (93.5)	
Independence	3374 (29.9)	3289 (32.9)	85 (6.5)	
Loneliness, n (%)				<.001
Yes	3581 (31.7)	2943 (29.4)	638 (49.2)	
No	7722 (68.3)	7062 (70.6)	660 (50.8)	
Smoking status, n (%)				<.001
Never smoked	7271 (64.3)	6230 (62.3)	1041 (80.2)	
Former or current smoker	4032 (35.7)	3775 (37.7)	257 (19.8)	
Drinking status, n (%)				<.001
Never drank	7670 (67.9)	6665 (66.6)	1005 (77.4)	
Former or current drinker	3633 (32.1)	3340 (33.4)	293 (22.6)	
Multimorbidity, n (%)				.02
Yes	1068 (9.4)	969 (9.7)	99 (7.6)	
No	10,235 (90.6)	9036 (90.3)	1199 (92.4)	
Sleep quality, n (%)				<.001
Good	7507 (66.4)	6706 (67.0)	801 (61.7)	
Poor	3796 (33.6)	3299 (33.0)	497 (38.3)	
Sleep duration, n (%)				<.001
Short (<6 h)	1327 (11.7)	1179 (11.8)	148 (11.4)	
Moderate (6‐9 h)	7207 (63.8)	6557 (65.5)	650 (50.1)	
Long (>9 h)	2769 (24.5)	2269 (22.7)	500 (38.5)	

a CF: cognitive frailty.

### Association Between Sleep Duration and Cognitive Frailty at Baseline

**Figure 2.**
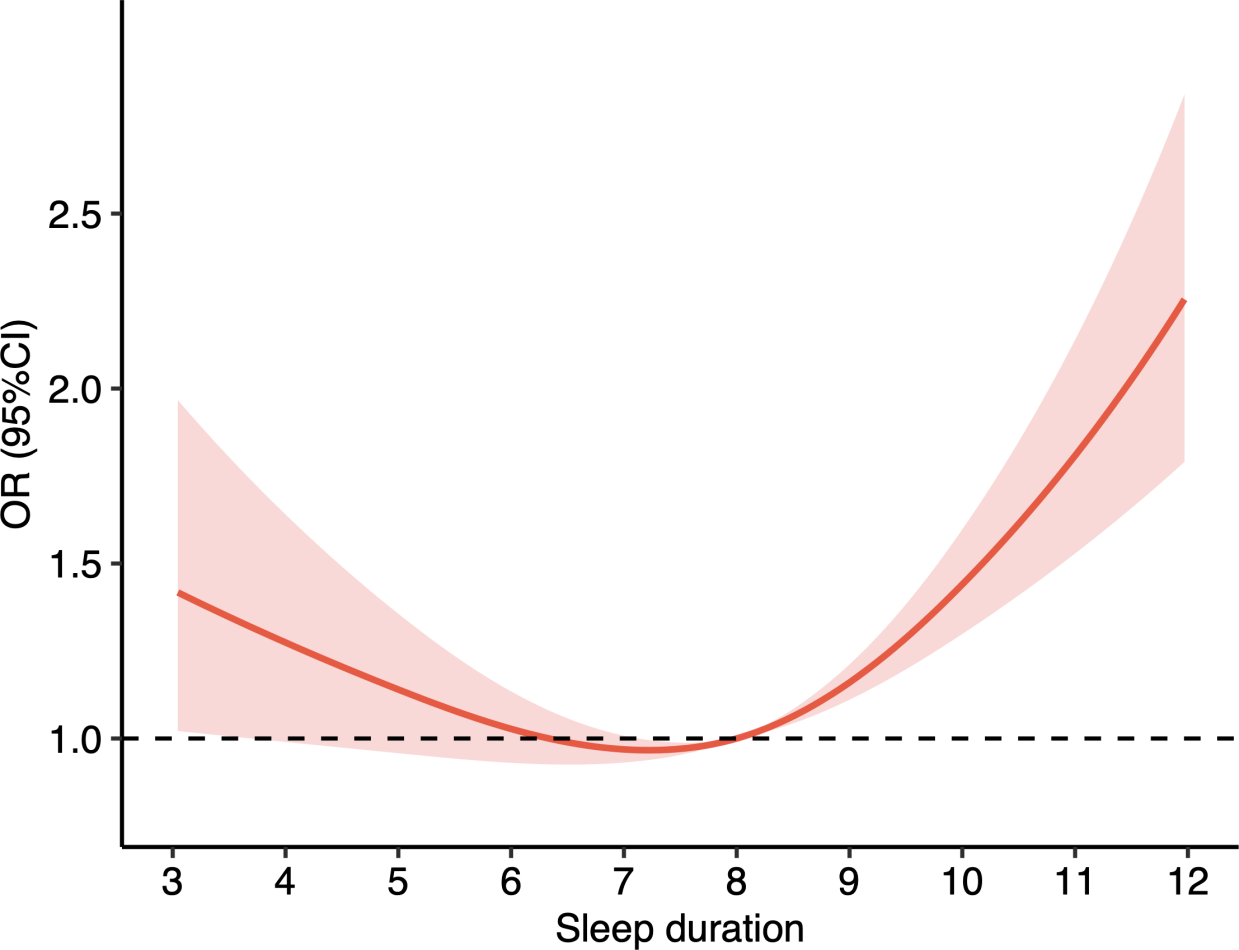
Nonlinear association between sleep duration and cognitive frailty at baseline using restricted cubic spline. The logistic regression model was used to fit the data, with solid lines representing the estimated OR and shaded areas representing the corresponding 95% CIs. OR: odds ratio.

Compared to participants who had moderate sleep duration, the odds of having cognitive frailty were higher in those who had long sleep duration (odds ratio [OR] 1.71, 95% CI 1.48‐1.97; *P*<.001) in the initial model adjusted for age, sex, and education Table 2. The association remained statistically significant with a similar OR in the augmented model after further adjustments of marital status, residence, economic status, loneliness, smoking status, drinking status, and multimorbidity (OR 1.69, 95% CI 1.46‐1.95; *P*<.001). There was no statistically significant difference in the odds of having cognitive frailty between participants with short and modest sleep durations in the initial model adjusted for age, sex, and education. Compared with those who had a good sleep quality, participants who had a poor sleep quality were at a higher risk for cognitive frailty in both initial and the augmented models with further adjustments (initial model: OR 1.63, 95% CI 1.40‐1.89; *P*<.001; augmented model: OR 1.41, 95% CI 1.21‐1.64; *P*<.001; Table 2). In addition, RCS analysis revealed a J-shaped association between sleep duration and cognitive frailty at baseline (*P* for nonlinear<.001; Figure 2).

The results of sensitivity analyses are summarized in MultimediaAppendices 3  4. The associations between long sleep duration and baseline cognitive frailty remained consistent when adjusting for each chronic disease individually and when accounting for all chronic diseases simultaneously (Multimedia Appendix 3). For individuals younger than 80 years old, the association of long sleep duration with cognitive frailty became not statistically significant, whereas for individuals older than 80 years old, the association persisted. The association remained significant in sex-stratified models (Multimedia Appendix 4).

**Table 2.** Association between sleep duration and cognitive frailty at baseline.

	Model A^a^		Model B^b^		Model C^c^	
	OR^d^ (95% CI)	*P* value	OR (95% CI)	*P* value	OR (95% CI)	*P* value
Age	—^e^	—	1.12 (1.12‐1.13)	<.001	1.12 (1.11‐1.13)	<.001
Sex (female)	—	—	1.80 (1.54‐2.11)	<.001	1.56 (1.30‐1.87)	<.001
Not educated	—	—	1.78 (1.49‐2.13)	<.001	1.57 (1.31‐1.90)	<.001
Married and living with spouse	—	—	—	—	1.21 (0.97‐1.52)	.09
Rural residence	—	—	—	—	0.86 (0.75‐0.99)	.04
Economic dependence	—	—	—	—	2.24 (1.75‐2.89)	<.001
Loneliness	—	—	—	—	1.82 (1.60‐2.09)	<.001
Smoker	—	—	—	—	0.91 (0.76‐1.10)	.33
Drinker	—	—	—	—	1.03 (0.87‐1.22)	.73
Multimorbidity	—	—	—	—	1.47 (1.14‐1.87)	.002
Poor sleep quality	1.62 (1.41‐1.85)	<.001	1.63 (1.40‐1.89)	<.001	1.41 (1.21‐1.64)	<.001
Sleep duration (hours)						
Moderate (6‐9)	reference	reference	reference	reference	reference	reference
Short (<6)	1.01 (0.82‐1.23)	.95	1.04 (0.83‐1.29)	.75	1.06 (0.85‐1.32)	.61
Long (>9)	2.47 (2.17-2.82)	<.001	1.71 (1.48‐1.97)	<.001	1.69 (1.46‐1.95)	<.001

a Model was unadjusted.

b Model was adjusted for age, sex, and education at baseline.

c Model was adjusted for age, sex, education, marital status, residence, economic status, loneliness, smoking status, drinking status, and multimorbidity at baseline.

d OR: odds ratio.

e Not applicable.

### Association Between Sleep Duration and Incident Cognitive Frailty During Follow-Up

**Figure 3.**
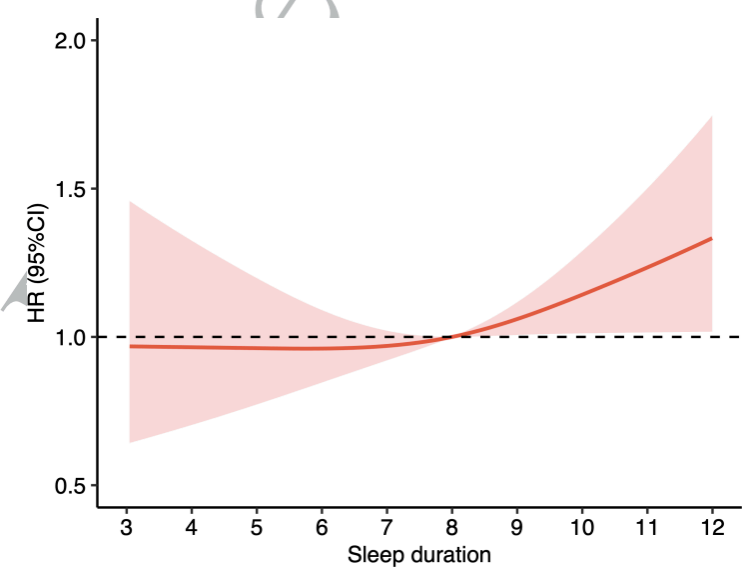
Nonlinear association between sleep duration and cognitive frailty during follow up using restricted cubic spline. The Cox proportional model was used to fit the data, with solid lines representing the estimated HR and shaded areas representing the corresponding 95% CIs. HR: hazard ratio.

Over a mean of 6.7 (SD 2.6) years of follow up, 521 (10%) participants out of the 5201 participants developed cognitive frailty. In the initial model adjusted for age, sex, and education, as compared with participants who had moderate sleep duration at baseline, a higher risk of cognitive frailty was observed in participants who had long sleep duration at baseline (hazard ratio [HR] 1.32, 95% CI 1.07‐1.62; *P*=.008; Table 3). The association persisted after further adjusting for marital status, residence, economic status, loneliness, smoking status, drinking status, and multimorbidity (HR 1.30, 95% CI 1.06‐1.59; *P*=.01). There was no statistically significant difference in the hazard of incident cognitive frailty between participants with short sleep duration and moderate sleep duration in the initial model. Besides, there was no significant difference in the risk of incident cognitive frailty between participants with good and poor sleep quality. Additionally, RCS analysis showed no significant nonlinear association between sleep duration and cognitive frailty during follow up (*P* for nonlinear=.29; Figure 3).

**Table 3.** Association between sleep duration and incident cognitive frailty during follow up.

	Model A^a^		Model B^b^		Model C^c^	
	HR^d^ (95% CI)	*P* value	HR (95% CI)	*P* value	HR (95% CI)	*P* value
Age	—^e^	—	1.09 (1.08‐1.10)	<.001	1.09 (1.08‐1.10)	<.001
Sex (female)	—	—	1.98 (1.60‐2.45)	<.001	1.61 (1.25‐2.08)	<.001
Not educated	—	—	1.84 (1.47‐2.31)	<.001	1.72 (1.36‐2.17)	<.001
Married and living with spouse	—	—	—	—	1.27 (1.01‐1.59)	.04
Rural residence	—	—	—	—	1.03 (0.86‐1.24)	.74
Economic dependence	—	—	—	—	1.47 (1.14‐1.91)	.003
Loneliness	—	—	—	—	1.22 (1.01‐1.47)	.04
Smoker	—	—	—	—	1.02 (0.80‐1.30)	.85
Drinker	—	—	—	—	0.77 (0.61‐0.97)	.03
Multimorbidity	—	—	—	—	1.33 (0.96‐1.83)	.08
Poor sleep quality	1.32 (1.08‐1.60)	.006	1.22 (1.00‐1.49)	.05	1.14 (0.93‐1.39)	.22
Sleep duration (hours)						
Moderate (6‐9)	reference	reference	reference	reference	reference	reference
Short (<6)	0.96 (0.72‐1.27)	.76	0.90 (0.68‐1.21)	.49	0.94 (0.70‐1.25)	.66
Long (>9)	1.74 (1.42‐2.14)	<.001	1.32 (1.07‐1.62)	.008	1.30 (1.06‐1.59)	.01

a Model was unadjusted.

b Model was adjusted for age, sex, and education at baseline.

c Model was adjusted for age, sex, education, marital status, residence, economic status, loneliness, smoking status, drinking status and multimorbidity at baseline.

d HR: hazard ratio.

e Not applicable.

The results of sensitivity analyses are summarized in MultimediaAppendices 5  6. The associations between long sleep duration and baseline cognitive frailty status were consistent when adjusting for each chronic disease individually, or when adjusting for all chronic diseases simultaneously (Multimedia Appendix 5). For individuals younger than 80 years old, the association of long sleep duration with cognitive frailty became not statistically significant, whereas for individuals older than 80 years old, the association persisted. The association was not significant in male participants but remained statistically significant in female participants (Multimedia Appendix 6).

### Rate of Change in Sleep Duration and Cognitive Frailty

The mean annual rate of change in sleep duration was –0.02 (SD 0.87) hours/year. In the model adjusted for age, sex, education, sleep quality, and sleep duration at baseline, a faster increase in sleep duration per year was associated with a higher risk for cognitive frailty: for 1 hour/year increase in sleep duration, the HR was 1.14 (95% CI 1.03‐1.27; *P*=.01). The association remained significant after further adjusting for marital status, residence, economic status, loneliness, smoking status, drinking status, and multimorbidity (HR 1.15, 95% CI 1.03‐1.27; *P*=.01; Multimedia Appendix 7).

## Discussion

### Principal Findings

In this large community-based prospective study of over 10,000 Chinese older adults, we found that long sleep duration was consistently associated with cognitive frailty status at baseline, as well as an increased risk of cognitive frailty incident during follow up. We also found that a faster annual increase in sleep duration was associated with a higher risk of cognitive frailty during follow up.

The associations of sleep duration with physical frailty or cognitive decline are complex with mixed findings in prior research. A study using data from the National Health and Nutrition Examination Survey demonstrated that prolonged sleep duration (ie, ≥10 h) was associated with physical frailty in older adults, while a prospective study of 309 older Mexican adults showed that participants with either short sleep duration (ie, ≤5 h) or long sleep duration (ie, ≥9 h) had a higher risk of physical frailty [22,23]. Likewise, a Japanese cohort study of 623 older adults reported that long sleepers (>8 h) had a higher risk of cognitive impairment, while a cohort study found that self-reported short sleepers (<7 h) had an increased risk of cognitive decline [24,25]. However, few studies explored the association between sleep duration and cognitive frailty, that is, the coexistence of physical frailty and cognitive impairment. Two recent cross-sectional studies reported an association of long sleep duration with cognitive frailty among community-dwelling older adults and in older adults with heart failure [11,12]. In this study, we confirmed such a cross-sectional association between long sleep duration and cognitive frailty. More intriguingly, we showed consistent evidence from longitudinal analyses that participants with long sleep duration at baseline had an increased risk of developing cognitive frailty in the future. Of note, we did not find a significant association between short sleep duration and cognitive frailty in this study.

We also used the annual rate of change in sleep duration to assess the relationship between sleep duration changes and cognitive frailty. The results showed that a faster annual increase in sleep duration was associated with a higher risk of cognitive frailty, independent of the baseline sleep duration category. Notably, this study accounted for variations in follow-up years by using the annual rate of sleep duration change instead of the absolute change in sleep duration, minimizing classification bias and providing a more accurate representation of the relationship between sleep duration trajectories and cognitive frailty.

The mechanisms underlying the association of sleep duration with incident cognitive frailty remain unclear. However, several potential pathways can be suggested. First, inappropriate sleep duration (either shorter or longer than ideal) has been linked to cardiovascular health [26], which has been reported as a risk factor for both physical frailty and cognitive decline [27,28]. Second, increased sleep duration has also been linked with elevations in C-reactive protein, a marker for systemic inflammation, and interleukin-6, a proinflammatory biomarker [29,30]. Prior studies have shown that even low-level inflammation may pose an increased risk for the development of physical frailty and cognitive impairment [31,32]. Finally, long sleep duration may also be an indicator of or linked to disturbances in circadian control, the internal biological clock that prepares bodily responses to environmental light-dark changes. Circadian disturbances have been previously reported to be associated with both physical frailty and cognitive impairment or decline [33-36].

Several previous studies showed that sleep quality is closely related to impairments in both physical and cognitive domains [37,38]. In addition, 2 recent cross-sectional studies reported that poor sleep quality was associated with cognitive frailty in older Chinese adults living in nursing homes, and among Thai community-dwelling older adults during the COVID-19 restrictions [39,40]. Our observed cross-sectional association between sleep quality and cognitive frailty aligns with these previous findings. However, we did not find evidence of an association between sleep quality and the development of cognitive frailty in this study. It is important to note that the assessment of sleep quality in this study relied on a single question, which may be overly simplistic and may not fully capture the complexity of sleep behaviors. Future studies should use more comprehensive approaches to assess sleep health, considering its multidimensional nature, which includes factors such as duration, timing, efficiency, satisfaction, and alertness [41].

### Strengths and Limitations

To the best of our knowledge, this study was the first to assess the association between sleep duration and incident cognitive frailty in a nationally representative sample of older Chinese adults. Strengths of this study include its longitudinal design and a large sample size. This study also has several limitations. First, the sleep duration and sleep quality were from self-reports and may be subjected to recall biases, especially in older adults and individuals with cognitive impairment. Future studies, ideally with an objective sleep monitoring approach (such as combining actigraphy and sleep diary or polysomnography), are warranted. Second, a growing body of studies have linked daytime napping behaviors with physical frailty, cognitive decline, Alzheimer dementia, or cognitive frailty [40,42,43]. Similarly, sleep disorders, such as sleep apnea, have been consistently linked with physical frailty, cognitive impairment, and dementia [10,44]. However, these potential sleep-related influences were not collected in the CLHLS. Future studies may consider incorporating these multidimensional structs when assessing sleep health and its relationships with physical and cognitive outcomes. Finally, the participants in this study were, on average, older than 80 years at baseline. In subgroup analyses, the association between long sleep duration and cognitive frailty was not statistically significant in participants younger than 80 years. This may be due to the limited number of cognitive frailty events in this subgroup, which could have resulted in insufficient statistical power to detect a significant association. Therefore, caution should be taken when translating our findings into younger populations.

### Conclusions

In this large cohort study of over 10,000 older Chinese adults, long sleep duration was consistently associated with an increased risk of cognitive frailty, both cross-sectionally and longitudinally. These findings underscore the importance of sleep monitoring in aging populations. Future research using objective sleep assessments and investigating the underlying biological mechanisms is warranted to inform more effective prevention strategies.

## Supplementary material

10.2196/65183 Multimedia Appendix 1 Baseline characteristics of participants by sleep duration groups

10.2196/65183 Multimedia Appendix 2 Baseline characteristics of participants by incident cognitive frailty status during follow-up.

10.2196/65183 Multimedia Appendix 3 Association between sleep and cognitive frailty at baseline with individual chronic disease adjustments.

10.2196/65183 Multimedia Appendix 4 Association between sleep and baseline cognitive frailty stratified by age and sex.

10.2196/65183 Multimedia Appendix 5 Association between sleep and incident cognitive frailty with individual chronic disease adjustments.

10.2196/65183 Multimedia Appendix 6 Association between sleep and incident cognitive frailty stratified by age and sex.

10.2196/65183 Multimedia Appendix 7 Rate of change in sleep duration and cognitive frailty.

## Abbreviations

C-MMSE: Chinese version of the Mini-Mental State Examination
CLHLS: Chinese Longitudinal Healthy Longevity Survey
HR: hazard ratio
OR: odds ratio
PH: proportional hazard
RCS: restricted cubic spline
