# Supplementary material for: Association Between Sleep Duration and Cognitive Frailty in Older Chinese Adults: Prospective Cohort Study
Source: JMIR Aging. 2025 Apr 23;8:e65183. doi: 10.2196/65183 (PMC12043274; doi:10.2196/65183)
Supplement: Multimedia Appendix 1 [file aging-v8-e65183-s001.docx]

|  | Short sleep duration (n=1327) | Moderate sleep duration (n=7207) | Long sleep duration (n=2769) | *p* value |
| --- | --- | --- | --- | --- |
| Age (years, mean ± SD) | 83.9 ± 10.1 | 83.4 ± 11.0 | 88.3 ± 10.4 | <0.001 |
| Sex |  |  |  | 0.001 |
| Male | 555 (41.8) | 3416 (47.4) | 1295 (46.8) |  |
| Female | 772 (58.2) | 3791 (52.6) | 1474 (53.2) |  |
| Education |  |  |  | <0.001 |
| Not educated | 811 (61.1) | 3976 (55.2) | 1761 (63.6) |  |
| With formal education | 516 (38.9) | 3231 (44.8) | 1008 (36.4) |  |
| Marital status |  |  |  | <0.001 |
| Married and living with spouse | 483 (36.4) | 2852 (39.6) | 763 (27.6) |  |
| Others | 844 (63.6) | 4355 (60.4) | 2006 (72.4) |  |
| Current residence |  |  |  | 0.016 |
| Urban | 514 (38.7) | 3034 (42.1) | 1099 (39.7) |  |
| Rural | 813 (61.3) | 4173 (57.9) | 1670 (60.3) |  |
| Economic status |  |  |  | <0.001 |
| Dependence | 960 (72.3) | 4806 (66.7) | 2163 (78.1) |  |
| Independence | 367 (27.7) | 2401 (33.3) | 606 (21.9) |  |
| Loneliness |  |  |  | <0.001 |
| Yes | 498 (37.5) | 2215 (30.7) | 868 (31.3) |  |
| No | 829 (62.5) | 4992 (69.3) | 1901 (68.7) |  |
| Smoking status |  |  |  | 0.199 |
| Never smoked | 883 (66.5) | 4618 (64.1) | 1770 (63.9) |  |
| Former or current smoker | 444 (33.5) | 2589 (35.9) | 999 (36.1) |  |
| Drinking status |  |  |  | 0.202 |
| Never drank | 925 (69.7) | 4892 (67.9) | 1853 (66.9) |  |
| Former or current drinker | 402 (30.3) | 2315 (32.1) | 916 (33.1) |  |
| Multimorbidity |  |  |  | <0.001 |
| Yes | 176 (13.3) | 664 (9.2) | 228 (8.2) |  |
| No | 1151 (86.7) | 6543 (90.8) | 2541 (91.8) |  |
| Sleep quality |  |  |  | <0.001 |
| Good | 1091 (82.2) | 4864 (67.5) | 2407 (86.9) |  |
| Poor | 236 (17.8) | 2343 (32.5) | 362 (13.1) |  |

Number (%) were reported.

SD, standard deviation.
