# Supplementary material for: Association Between Sleep Duration and Cognitive Frailty in Older Chinese Adults: Prospective Cohort Study
Source: JMIR Aging. 2025 Apr 23;8:e65183. doi: 10.2196/65183 (PMC12043274; doi:10.2196/65183)
Supplement: Multimedia Appendix 2 [file aging-v8-e65183-s002.docx]

|  | Overall  (n=5201) | Non-CF  (n=4680) | CF  (n=521) | *p* value |
| --- | --- | --- | --- | --- |
| Age (years, mean ± SD) | 79.1 ± 9.5 | 78.3 ± 9.2 | 86.5 ± 8.9 | <0.001 |
| Sex |  |  |  | <0.001 |
| Male | 2629 (50.6) | 2490 (53.2) | 138 (23.7) |  |
| Female | 2572 (49.5) | 2190 (46.8) | 382 (73.3) |  |
| Education |  |  |  | <0.001 |
| Not educated | 2585 (49.7) | 2183 (46.7) | 402 (77.2) |  |
| With formal education | 2616 (50.3) | 2497 (53.4) | 119 (22.8) |  |
| Marital status |  |  |  | <0.001 |
| Married and living with spouse | 2638 (50.7) | 2506 (53.6) | 132 (25.3) |  |
| Others | 2563 (49.3) | 2174 (46.5) | 389 (74.7) |  |
| Current residence |  |  |  | 0.010 |
| Urban | 2043 (39.3) | 1866 (39.9) | 177 (34.0) |  |
| Rural | 3158 (60.7) | 2814 (60.1) | 344 (66.0) |  |
| Economic status |  |  |  | <0.001 |
| Dependence | 3185 (61.2) | 2748 (58.7) | 437 (83.9) |  |
| Independence | 2016 (38.8) | 1932 (41.3) | 84 (16.1) |  |
| Loneliness |  |  |  | <0.001 |
| Yes | 1282 (24.7) | 1102 (23.6) | 180 (34.6) |  |
| No | 3919 (75.4) | 3578 (76.5) | 341 (65.5) |  |
| Smoking status |  |  |  | <0.001 |
| Never smoked | 3153 (60.6) | 2756 (58.9) | 397 (76.2) |  |
| Former or current smoker | 2048 (39.4) | 1924 (41.1) | 124 (23.8) |  |
| Drinking status |  |  |  | <0.001 |
| Never drank | 3380 (65.0) | 2974 (63.5) | 406 (77.9) |  |
| Former or current drinker | 1821 (35.0) | 1706 (36.5) | 115 (22.1) |  |
| Multimorbidity |  |  |  | 0.229 |
| Yes | 501 (9.6) | 459 (9.8) | 42 (8.1) |  |
| No | 4700 (90.4) | 4221 (90.2) | 479 (91.9) |  |
| Sleep quality |  |  |  | 0.070 |
| Good | 3493 (67.2) | 3162 (67.6) | 331 (63.5) |  |
| Poor | 1708 (32.8) | 1518 (32.4) | 190 (36.5) |  |
| Sleep duration |  |  |  | <0.001 |
| Short (< 6 h) | 638 (12.3) | 575 (12.3) | 63 (12.1) |  |
| Moderate (6-9 h) | 3563 (68.5) | 3242 (69.3) | 321 (61.6) |  |
| Long (> 9 h) | 1000 (19.2) | 863 (18.4) | 137 (26.3) |  |

Number (%) were reported.

CF: Cognitive frailty; SD, standard deviation.
