# Supplementary material for: Association Between Sleep Duration and Cognitive Frailty in Older Chinese Adults: Prospective Cohort Study
Source: JMIR Aging. 2025 Apr 23;8:e65183. doi: 10.2196/65183 (PMC12043274; doi:10.2196/65183)
Supplement: Multimedia Appendix 3 [file aging-v8-e65183-s003.docx]

|  | OR (95% CI) | *p* value |
| --- | --- | --- |
| *Hypertension* |  |  |
| Poor sleep quality | 1.42 (1.22-1.65) | <0.001 |
| Short sleep duration (< 6 h) | 1.05 (0.84-1.31) | 0.641 |
| Long sleep duration (> 9 h) | 1.69 (1.46-1.95) | <0.001 |
| *Diabetes* |  |  |
| Poor sleep quality | 1.42 (1.22-1.65) | <0.001 |
| Short sleep duration (< 6 h) | 1.06 (0.85-1.31) | 0.627 |
| Long sleep duration (> 9 h) | 1.69 (1.46-1.95) | <0.001 |
| *Heart disease* |  |  |
| Poor sleep quality | 1.42 (1.22-1.65) | <0.001 |
| Short sleep duration (< 6 h) | 1.06 (0.85-1.31) | 0.630 |
| Long sleep duration (> 9 h) | 1.69 (1.46-1.96) | <0.001 |
| *Stroke and cerebrovascular disease* |  |  |
| Poor sleep quality | 1.41 (1.21-1.64) | <0.001 |
| Short sleep duration (< 6 h) | 1.05 (0.84-1.31) | 0.664 |
| Long sleep duration (> 9 h) | 1.69 (1.46-1.95) | <0.001 |
| *Respiratory disease* |  |  |
| Poor sleep quality | 1.43 (1.22-1.66) | <0.001 |
| Short sleep duration (< 6 h) | 1.05 (0.84-1.31) | 0.647 |
| Long sleep duration (> 9 h) | 1.69 (1.46-1.95) | <0.001 |
| *Cancer* |  |  |
| Poor sleep quality | 1.43 (1.23-1.66) | <0.001 |
| Short sleep duration (< 6 h) | 1.05 (0.84-1.31) | 0.639 |
| Long sleep duration (> 9 h) | 1.69 (1.46-1.95) | <0.001 |
| *Parkinson's disease* |  |  |
| Poor sleep quality | 1.43 (1.23-1.67) | <0.001 |
| Short sleep duration (< 6 h) | 1.05 (0.84-1.31) | 0.641 |
| Long sleep duration (> 9 h) | 1.69 (1.46-1.95) | <0.001 |
| *All diseases* |  |  |
| Poor sleep quality | 1.39 (1.19-1.62) | <0.001 |
| Short sleep duration (< 6 h) | 1.05 (0.84-1.30) | 0.680 |
| Long sleep duration (> 9 h) | 1.69 (1.46-1.95) | <0.001 |

All models were further adjusted for age, sex, education, marital status, residence, economic status, loneliness, smoking status, and drinking status at baseline.

OR: odds ratio; CI: confidence interval.
