# Supplementary material for: Association Between Sleep Duration and Cognitive Frailty in Older Chinese Adults: Prospective Cohort Study
Source: JMIR Aging. 2025 Apr 23;8:e65183. doi: 10.2196/65183 (PMC12043274; doi:10.2196/65183)
Supplement: Multimedia Appendix 4 [file aging-v8-e65183-s004.docx]

|  | OR (95% CI) ^a^ | *p* value ^a^ | OR (95% CI) ^b^ | *p* value ^b^ |
| --- | --- | --- | --- | --- |
| *Stratifying participants by age at baseline* |  |  |  |  |
| <80 years old (n=3770, CF=29) |  |  |  |  |
| Poor sleep quality | 2.00 (0.87-4.58) | 0.100 | 1.81 (0.79-4.16) | 0.157 |
| Short sleep duration (< 6 h) | 1.60 (0.57-4.13) | 0.347 | 1.48 (0.53-3.84) | 0.432 |
| Long sleep duration (> 9 h) | 1.79 (0.63-4.50) | 0.235 | 1.76 (0.61-4.45) | 0.256 |
| ≥80 years old (n=7533, CF=1269) |  |  |  |  |
| Poor sleep quality | 1.59 (1.37-1.85) | <0.001 | 1.38 (1.19-1.61) | <0.001 |
| Short sleep duration (< 6 h) | 1.00 (0.80-1.25) | 0.992 | 1.03 (0.82-1.28) | 0.806 |
| Long sleep duration (> 9 h) | 1.68 (1.45-1.94) | <0.001 | 1.67 (1.44-1.93) | <0.001 |
| *Stratifying participants by sex ^c^* |  |  |  |  |
| Female (n=6037, CF=998) |  |  |  |  |
| Poor sleep quality | 1.36 (1.14-1.61) | 0.001 | 1.20 (1.01-1.44) | 0.040 |
| Short sleep duration (< 6 h) | 1.19 (0.93-1.53) | 0.165 | 1.21 (0.94-1.55) | 0.137 |
| Long sleep duration (> 9 h) | 1.72 (1.45-2.03) | <0.001 | 1.70 (1.44-2.02) | <0.001 |
| Male (n=5266, CF=300) |  |  |  |  |
| Poor sleep quality | 2.61 (1.97-3.46) | <0.001 | 2.14 (1.60-2.86) | <0.001 |
| Short sleep duration (< 6 h) | 0.69 (0.43-1.08) | 0.116 | 0.73 (0.45-1.14) | 0.175 |
| Long sleep duration (> 9 h) | 1.70 (1.28-2.24) | <0.001 | 1.68 (1.26-2.22) | <0.001 |

^a^ Models were adjusted for age, sex, and education at baseline.

^b^ Models were adjusted for age, sex, education, marital status, residence, economic status, loneliness, smoking status, drinking status and multimorbidity at baseline.

^c^ In sex-stratified analyses, sex was not included as an adjustment variable.

OR: odds ratio; CI: confidence interval.
