# Supplementary material for: Association Between Sleep Duration and Cognitive Frailty in Older Chinese Adults: Prospective Cohort Study
Source: JMIR Aging. 2025 Apr 23;8:e65183. doi: 10.2196/65183 (PMC12043274; doi:10.2196/65183)
Supplement: Multimedia Appendix 5 [file aging-v8-e65183-s005.docx]

|  | HR (95% CI) | *p* value |
| --- | --- | --- |
| *Hypertension* |  |  |
| Poor sleep quality | 1.14 (0.93-1.40) | 0.195 |
| Short sleep duration (< 6 h) | 0.94 (0.71-1.26) | 0.695 |
| Long sleep duration (> 9 h) | 1.30 (1.06-1.59) | 0.013 |
| *Diabetes* |  |  |
| Poor sleep quality | 1.14 (0.93-1.39) | 0.209 |
| Short sleep duration (< 6 h) | 0.94 (0.71-1.26) | 0.697 |
| Long sleep duration (> 9 h) | 1.30 (1.06-1.59) | 0.013 |
| *Heart disease* |  |  |
| Poor sleep quality | 1.14 (0.93-1.39) | 0.205 |
| Short sleep duration (< 6 h) | 0.94 (0.70-1.25) | 0.660 |
| Long sleep duration (> 9 h) | 1.30 (1.06-1.60) | 0.012 |
| *Stroke and cerebrovascular disease* |  |  |
| Poor sleep quality | 1.15 (0.94-1.40) | 0.184 |
| Short sleep duration (< 6 h) | 0.92 (0.69-1.23) | 0.572 |
| Long sleep duration (> 9 h) | 1.31 (1.06-1.60) | 0.011 |
| *Respiratory disease* |  |  |
| Poor sleep quality | 1.14 (0.93-1.40) | 0.196 |
| Short sleep duration (< 6 h) | 0.94 (0.71-1.26) | 0.693 |
| Long sleep duration (> 9 h) | 1.30 (1.06-1.59) | 0.013 |
| *Cancer* |  |  |
| Poor sleep quality | 1.14 (0.93-1.40) | 0.192 |
| Short sleep duration (< 6 h) | 0.94 (0.70-1.25) | 0.673 |
| Long sleep duration (> 9 h) | 1.30 (1.06-1.59) | 0.013 |
| *Parkinson's disease* |  |  |
| Poor sleep quality | 1.14 (0.94-1.40) | 0.191 |
| Short sleep duration (< 6 h) | 0.94 (0.71-1.26) | 0.701 |
| Long sleep duration (> 9 h) | 1.31 (1.07-1.61) | 0.010 |
| *All diseases* |  |  |
| Poor sleep quality | 1.15 (0.94-1.40) | 0.180 |
| Short sleep duration (< 6 h) | 0.92 (0.69-1.22) | 0.556 |
| Long sleep duration (> 9 h) | 1.32 (1.08-1.63) | 0.007 |

All models were further adjusted for age, sex, education, marital status, residence, economic status, loneliness, smoking status, and drinking status at baseline.

HR: hazard ratio; CI: confidence interval.
