# Supplementary material for: Association Between Sleep Duration and Cognitive Frailty in Older Chinese Adults: Prospective Cohort Study
Source: JMIR Aging. 2025 Apr 23;8:e65183. doi: 10.2196/65183 (PMC12043274; doi:10.2196/65183)
Supplement: Multimedia Appendix 6 [file aging-v8-e65183-s006.docx]

|  | HR (95% CI) ^a^ | *p* value ^a^ | HR (95% CI) ^b^ | *p* value ^b^ |
| --- | --- | --- | --- | --- |
| *Stratifying participants by age at baseline* |  |  |  |  |
| <80 years old (n=2764, CF=111) |  |  |  |  |
| Poor sleep quality | 1.15 (0.76-1.75) | 0.513 | 1.12 (0.73-1.70) | 0.610 |
| Short sleep duration (< 6 h) | 0.96 (0.54-1.69) | 0.877 | 0.94 (0.53-1.66) | 0.833 |
| Long sleep duration (> 9 h) | 0.51 (0.25-1.01) | 0.053 | 0.50 (0.25-1.00) | 0.051 |
| ≥80 years old (n=2437, CF=410) |  |  |  |  |
| Poor sleep quality | 1.20 (0.96-1.50) | 0.117 | 1.12 (0.89-1.41) | 0.345 |
| Short sleep duration (< 6 h) | 0.87 (0.62-1.22) | 0.423 | 0.92 (0.66-1.29) | 0.639 |
| Long sleep duration (> 9 h) | 1.48 (1.18-1.84) | 0.001 | 1.47 (1.18-1.83) | 0.001 |
| *Stratifying participants by sex ^c^* |  |  |  |  |
| Female (n=2572, CF=382) |  |  |  |  |
| Poor sleep quality | 1.16 (0.93-1.46) | 0.197 | 1.09 (0.87-1.38) | 0.444 |
| Short sleep duration (< 6 h) | 0.78 (0.55-1.10) | 0.162 | 0.83 (0.58-1.17) | 0.286 |
| Long sleep duration (> 9 h) | 1.27 (1.00-1.62) | 0.048 | 1.25 (0.98-1.59) | 0.070 |
| *Male (n=2629, CF=139)* |  |  |  |  |
| Poor sleep quality | 1.36 (0.91-2.04) | 0.130 | 1.23 (0.82-1.85) | 0.320 |
| Short sleep duration (< 6 h) | 1.31 (0.77-2.22) | 0.313 | 1.28 (0.76-2.17) | 0.357 |
| Long sleep duration (> 9 h) | 1.46 (0.98-2.16) | 0.063 | 1.43 (0.97-2.13) | 0.073 |

^a^ Models were adjusted for age, sex, and education at baseline.

^b^ Models were adjusted for age, sex, education, marital status, residence, economic status, loneliness, smoking status, drinking status and multimorbidity at baseline.

^c^ In sex-stratified analyses, sex was not included as an adjustment variable.

HR: hazard ratio; CI: confidence interval.
