# Supplementary material for: Association Between Sleep Duration and Cognitive Frailty in Older Chinese Adults: Prospective Cohort Study
Source: JMIR Aging. 2025 Apr 23;8:e65183. doi: 10.2196/65183 (PMC12043274; doi:10.2196/65183)
Supplement: Multimedia Appendix 7 [file aging-v8-e65183-s007.docx]

|  | HR (95% CI) *p* value ^a^ | HR (95% CI) *p* value ^b^ | HR (95% CI) *p* value ^c^ |
| --- | --- | --- | --- |
| Age |  | 1.09 (1.08-1.10) <0.001 | 1.08 (1.07-1.10) <0.001 |
| Female sex |  | 1.97 (1.59-2.44) <0.001 | 1.61 (1.25-2.08) <0.001 |
| Not educated |  | 1.86 (1.48-2.34) <0.001 | 1.74 (1.38-2.19) <0.001 |
| Married and living with spouse |  |  | 1.27 (1.01-1.59) 0.039 |
| Rural residence |  |  | 1.03 (0.86-1.25) 0.721 |
| Economic dependence |  |  | 1.47 (1.14-1.90) 0.003 |
| Loneliness |  |  | 1.22 (1.01-1.47) 0.035 |
| Smoker |  |  | 1.03 (0.80-1.31) 0.840 |
| Drinker |  |  | 0.77 (0.61-0.97) 0.028 |
| Multimorbidity |  |  | 1.34 (0.97-1.85) 0.074 |
| Poor sleep quality |  | 1.20 (0.98-1.46) 0.072 | 1.12 (0.91-1.37) 0.285 |
| Sleep duration |  |  |  |
| Moderate (6-9 h) |  | Reference | Reference |
| Short (< 6 h) |  | 0.83 (0.62-1.12) 0.222 | 0.86 (0.64-1.16) 0.317 |
| Long (> 9 h) |  | 1.46 (1.17-1.82) <0.001 | 1.44 (1.16-1.80) 0.001 |
| Rate of change in sleep duration | 1.15 (1.04-1.27) 0.008 | 1.14 (1.03-1.27) 0.012 | 1.15 (1.03-1.27) 0.010 |

^a^ Model was unadjusted.

^b^ Model was adjusted for age, sex, education, sleep quality, and sleep duration at baseline.

^c^ Model was adjusted for age, sex, education, marital status, residence, economic status, loneliness, smoking status, drinking status, multimorbidity, sleep quality, and sleep duration at baseline.

HR: hazard ratio; CI: confidence interval.
